# Cannabis Legalization and Acute Harm From High Potency Cannabis Products: A Narrative Review and Recommendations for Public Health

**DOI:** 10.3389/fpsyt.2020.591979

**Published:** 2020-09-23

**Authors:** Justin Matheson, Bernard Le Foll

**Affiliations:** ^1^Translational Addiction Research Laboratory, Centre for Addiction and Mental Health, Toronto, ON, Canada; ^2^Department of Pharmacology and Toxicology, Faculty of Medicine, University of Toronto, Toronto, ON, Canada; ^3^Addictions Division, Centre for Addiction and Mental Health, Toronto, ON, Canada; ^4^Campbell Family Mental Health Research Institute, Centre for Addiction and Mental Health, Toronto, ON, Canada; ^5^Department of Psychiatry, Faculty of Medicine, University of Toronto, Toronto, ON, Canada; ^6^Institute of Medical Sciences, University of Toronto, Toronto, ON, Canada; ^7^Department of Family and Community Medicine, University of Toronto, Toronto, ON, Canada

**Keywords:** cannabis, legalization, acute harms, edibles, cannabis concentrates

## Abstract

Legalization and commercial sale of non-medical cannabis has led to increasing diversity and potency of cannabis products. Some of the American states that were the first to legalize have seen rises in acute harms associated with cannabis use, e.g. Colorado has seen increases in emergency department visits for cannabis-related acute psychological distress and severe vomiting (hyperemesis), as well as a number of high-profile deaths related to ingestion of high doses of cannabis edibles. Over-ingestion of cannabis is related to multiple factors, including the sale of cannabis products with high levels of THC and consumers’ confusion regarding labelling of cannabis products, which disproportionately impact new or inexperienced users. Based on our review of the literature, we propose three approaches to minimizing acute harms: early restriction of cannabis edibles and high-potency products; clear and consistent labelling that communicates dose/serving size and health risks; and implementation of robust data collection frameworks to monitor harms, broken down by cannabis product type (e.g. dose, potency, route of administration) and consumer characteristics (e.g. age, sex, gender, ethnicity). Ongoing data collection and monitoring of harms in jurisdictions that have existing legal cannabis laws will be vital to understanding the impact of cannabis legalization and maximizing public health benefits.

## Introduction

Cannabis continues to be one of the most commonly used psychoactive drugs worldwide, with recent estimates from the United Nations Office on Drugs and Crime (UNODC) suggesting over 188 million past-year users in 2017 (1). Cannabis has remained an illicit drug under international drug control treaties (in particular, the 1961 UN Single Convention on Narcotic Drugs), yet critics have opposed the criminalization of cannabis for a multitude of reasons since at least the 1960s (2, 3). For example, cannabis use is prevalent among young adults, yet is associated with less harm than licit drugs such as alcohol and tobacco (4, 5). Criminalization of cannabis use and possession has likely done more harm than good by exposing users to the criminal justice system (6), which has disproportionately affected disadvantaged minorities populations, especially Black and Hispanic communities (7). Legalizing cannabis has the potential to restore justice to by expunging arrests and by using taxes generated by the cannabis retail market to help rebuild these communities (7). Eliminating the illicit cannabis market would greatly reduce costs associated with policing of cannabis prohibition (6). Finally, having a legal retail market would allow for better control and regulation of cannabis products, e.g. by restricting access to youth and by protecting adult users from contaminants (e.g. fungi and heavy metals) and unsafe levels of Δ^9^-tetrahydrocannabinol (THC) (6).

In 2012, Colorado and Washington became the first two US states to pass referenda to legalize possession and retail sales of non-medical cannabis, with retail sales available in 2014 (6). At the time of this writing, 11 US states and the District of Columbia have legalized non-medical use and sale of cannabis, though cannabis remains illegal federally. In 2013, Uruguay became the first country to legalize at the federal level, using a middle-ground approach that involved more restrictions than the US legal retail markets (8). This was followed by the October 2018 federal legalization in Canada, where a regulated retail market was implemented (9), with similar legislation planned in countries such as Luxembourg and Mexico.

While there is potential for a net beneficial effect of legalization of non-medical cannabis use, concerns have arisen regarding increasing public health harms. Due to challenges in conducting epidemiological research (e.g. because of the legal status of cannabis and that most cannabis users worldwide also smoke tobacco), the adverse physical health effects of cannabis remain largely uncertain (2). One consistent finding has been an association between heavy, long-term use of cannabis and respiratory problems such as chronic bronchitis (10, 11). Limited evidence suggests cannabis use may elevate risk of cardiovascular disease (12, 13) and possibly testicular cancer (14). Cannabis hyperemesis syndrome (CHS) has emerged recently as a significant risk of chronic cannabis use. CHS is described as a paradoxical side effect of cannabis use (since cannabis has anti-emetic effects), and is characterized by cyclical nausea, vomiting, and abdominal pain with no clear etiology (15), though is thought to be related to changes in the endocannabinoid system and subsequent dysregulation of stress and anxiety responses (16, 17).

The long-term psychological adverse effects of regular cannabis use have been much more clearly demonstrated, though establishing causality remains an issue (2, 6, 18). Decline in cognitive function resulting from regular, heavy cannabis use has been robustly demonstrated in cross-sectional studies, prompting concerns about impairments in psychosocial functioning and educational/vocational attainment (19–21). Since the late 1980’s, at least a dozen prospective longitudinal studies have documented an association between cannabis use and increased risk of subsequent psychotic symptoms or illness (22). The association between cannabis and psychosis risk has been supported by compelling evidence from animal, human laboratory, and clinical studies (22–24). A subset of cannabis users will go on to develop a cannabis use disorder (CUD), which is an amalgamation of the diagnostic terms cannabis dependence and cannabis abuse that were used prior to the 5^th^ edition of the Diagnostic and Statistical Manual of Mental Disorders (DSM-V). Early evidence in the US suggested that about 1 in 10 people who use cannabis will develop cannabis dependence, which is lower than the conversion rates for tobacco, alcohol, cocaine, or heroin (25). A recent meta-analysis of 21 epidemiological studies conducted between 2009 and 2019 found that the risk of CUD among people who used cannabis was 22% (26). In 2012, CUDs were determined to be the leading cause of cannabis-attributable burden of disease in Canada (27). Cannabis use has also been associated with increased risk or exacerbation of other mental health problems such as anxiety and depression, though the relationship between cannabis use and mental health is complex (18, 28).

Compared to the chronic or long-term harms associated with cannabis use, acute harms have received less attention. Due to acute effects on cognitive performance, cannabis use has been associated with increased risk of motor vehicle collisions (29). Road traffic injuries were the leading cause of cannabis-attributable mortality among Canadians aged 45 years or younger in 2012 (27). Cannabis is not associated with overdose mortality, which is likely due to low risk of respiratory depression as a result of low or absent expression of cannabinoid receptors in the brainstem (30). However, a small number of deaths from cardiovascular events (13) or from hyperemesis syndrome (31) have been attributed to cannabis. In addition, the psychological consequences of acute cannabis intoxication (e.g. psychosis, suicidality, impairment-related injuries) can lead to emergency department (ED) visits and hospitalizations (32). ED visits have been linked to so-called “unexpected highs” that can occur when individuals consume more cannabis than intended (33).

While these acute harms remain low compared to harms associated with alcohol and illicit drugs such as heroin and methamphetamine, the emergence of the legal cannabis retail market has the potential to increase harms by increasing the potency and diversity of cannabis products, encouraging existing users to increase their quantity and frequency of use, and attracting new users who are unfamiliar with cannabis and may unintentionally ingest large doses. Thus, in this review, we discuss the risk of an increase in acute cannabis-related harms as legal retail cannabis markets emerge and proliferate, and then provide recommendations to public health based on evidence from states and countries that have already legalized non-medical cannabis.

## Diversification of Cannabis Products and the Potential Rise in Acute Harms: A Brief Review

As legal cannabis retail markets have emerged in the US, Canada, and Europe, the products available to consumers have changed dramatically over the past decade. Two particular changes have had the biggest impact: the increasing THC potency of cannabis and the diversity of cannabis products available.

One of the most important metrics of cannabis consumption is potency, which is typically quantified as the proportion of THC in a cannabis product. Cannabis potency estimates can also include levels of cannabidiol (CBD), a non-intoxicating cannabinoid that has been demonstrated to offset or reduce the negative impact of THC on anxiety, cognition, and psychotic symptoms (34, 35). While cannabis with higher proportions of THC is generally regarded as more harmful, increasing levels of CBD in cannabis may reduce harms (35). In 2012, a meta-analysis of 75 individual estimates of THC potency worldwide found a striking 10-fold increase in THC potency of dried plant material between 1970 and 2009 (36). In England, the potency of sinsemilla (unpollinated female flower) doubled between 1995 and 2005 (37), though did not change considerably between 2005 and 2016 (38). More recently, in the United States, mean THC potency increased from 8.9% in 2008 to 17.1% in 2017, while the THC:CBD ratio increased dramatically from 23 in 2008 to 104 in 2017 (39). In Europe, mean herbal cannabis potency increased from 5% in 2006 to 10% in 2016 (40). Recent estimates in the Canadian market suggest similar (or greater) increases in the potency of cannabis. For example, one study that tracked the potency of legal and illegal cannabis products for two months following the federal legalization of non-medical cannabis use found a mean THC concentration of 16.1% in the legal market and 20.5% in the illegal market (41). Given that the global mean THC potency of cannabis was approximately 1–2% just a few decades ago (36), the emergence of dried cannabis plant material with 20% THC or more presents a serious public health concern, especially in the absence of a proportional increase in CBD levels.

While smoking dried cannabis flower has historically been the dominant method of cannabis use, the emergence of a legal retail market has led to unprecedented diversification of cannabis products and formulations, driven by both increasing popularity of less common methods of use and the creation of entirely new products (42). An existing method of cannabis use that has been gaining popularity is the ingestion of cannabis edibles, which are typically desserts that use cannabis-infused oil in the baking process (43). In addition to traditional cannabis edibles (i.e., baked goods), other oral THC products such as THC-infused candies and other foodstuffs, oils, and tinctures have become common in legal retail markets (42). The use of edible cannabis products may be preferred by medical or non-medical users who do not want to be exposed to cannabis smoke (44), and edibles have been suggested to reduce the respiratory risks associated with combustible cannabis use (10). However, one major concern with the use of edibles is the delayed and often unpredictable onset and duration of psychotropic effects as a result of the slower absorption of THC into the systemic circulation (45, 46). A recent survey of adult past-year users of cannabis in Colorado found that use of edibles was associated with greater odds of experiencing an unexpected high (33). A second existing method of cannabis use rising in popularity is vaporized cannabis (42). Vaping devices typically operate at temperatures that do not combust the cannabis product, but rather aerosolize cannabinoids for inhalation, which likely exposes the user to fewer toxicants (42). However, concerns about vaping have arisen as a result of recent injuries and deaths associated with use of vaporizers, such as the series of 98 cases of lung injury in Wisconsin and Illinois documented in 2019 (47).

Several newer trends of cannabis use have emerged, such as the combustion and inhalation of cannabis concentrates (e.g. waxes, “dabs”, and “shatter”) (42, 48). These products often have very high concentrations of THC, are commonly used for their greater drug-induced “high”, and have been associated with a number of acute harms (42). For example, “dabs” are concentrated extracts of hashish oil created using a butane solvent, while “dabbing” refers to the behavior of heating the extract on a device and inhaling the resulting vapor, often resulting in a very large and immediate dose of THC (49). The use of “dabs” has been associated with cases of acute psychosis, cardiotoxicity, and respiratory failure, though the exact causality remains unknown (49). The use of cannabis concentrates in vaporizers has been associated with increased risk of pulmonary injury and other acute harms (50). In addition to cannabis concentrates, a recent plethora of diverse products have emerged, such as topicals (lotions, balms, creams, etc.), sublingual sprays, and even rectal and vaginal suppositories (42). Very little is known about these new cannabis products. In addition to cannabis-derived products, synthetic cannabinoids have risen in popularity recently, which is concerning given their significant association with severe adverse health effects and deaths (51, 52). While these compounds are unlikely to be marketed along with cannabis products in a legal retail market, it will be important to monitor their use as attitudes toward cannabinoid products change.

Evidence in the US has demonstrated a relationship between specific provisions in legal cannabis laws (both medical and non-medical laws) and an increase in likelihood of using alternative methods of cannabis among youths, especially edibles and vaping (53, 54). Similarly, cannabis laws that permit home cultivation were found to increase the odds of individuals making cannabis edibles at home, while laws permitting cannabis dispensaries increased the odds of purchasing edibles (55).

The increase in potency and diversity of cannabis products is concerning as it challenges the generalizability of previous studies of acute cannabis-related harms. For example, the acute effects of THC in human laboratory studies are often dose-dependent (18, 19), yet research conducted in the United States is limited to using cannabis produced by the National Institute on Drug Abuse (NIDA), which was found to be nearly one quarter of the potency of cannabis available in retail markets (56). Similarly, the majority of placebo-controlled studies of acute effects have administered dried flower by the smoked route, while very few studies have assessed the effects of edibles, and virtually no controlled studies have assessed the acute effects of newer products like concentrates, tinctures, or oils (42). Epidemiological studies have also documented associations between higher-potency cannabis and increased risk of CUD (57) and psychosis (58), though specific associations with acute harms are less clear.

Data that allow for monitoring of changes in acute cannabis-related harms after the emergence of legal retail markets are scarce, as most jurisdictions have only a few years of data since legalization, and the scope and quality of data collection varies. Most evidence for rises in acute harms have relied on hospitalization data. Colorado state in particular has a long history of liberalization of cannabis attitudes and legislation, which, along with wider availability of cannabis, has led to greater longitudinal availability of data to describe patterns in cannabis use and related harms (59). After legalization of non-medical cannabis use in 2012 and opening of retail sales in early 2014, Colorado saw significant evidence of increasing acute harms, including increases in cannabis-related ED visits and accidental poisonings in young children, as well as a handful of deaths related to consumption of cannabis edibles (32, 59, 60).

A recent chart review of adult visits to a large academic hospital in Colorado between January 2012 and December 2016 found that gastrointestinal symptoms, acute intoxication, and psychiatric symptoms were the three most common reasons for cannabis-attributable visits to the ED (61). While visits attributable to inhaled cannabis were more common overall, visits attributable to edible cannabis were more likely to be a result of acute psychiatric symptoms and intoxication (61). Importantly, the number of ED visits at least partially attributable to cannabis significantly increased from 2012 to 2016 (62). Other ED data have similarly found increases in cannabis-attributable visits from pre- to post-legalization, especially relating to mental health (63, 64), and have specifically seen an increase in adolescent cannabis-related ED visits (65). There was a significant increase in the proportion of suicide victims who tested positive for cannabis, from 7.1% in 2004–2009 to 12.6% in 2010–2015 (32). An analysis of hospital admissions in Colorado between 2010 and 2014 found a significant increase in hospitalizations related to cyclical vomiting (66), suggesting an increase in CHS. Other data suggest an increase in the age of patients presenting with skull fractures following legalization (which was suggested to be a result of increased use of cannabis among older patients) (67), and an increase in detection of cannabis in patients presenting to Colorado hospitals with traumatic injuries (68). In addition, legalization of non-medical cannabis use in Colorado (but not Washington state) was associated with an increase in traffic fatalities (69).

Limited data on hospitalizations associated with cannabis are available in Canada as well. For example, data collected in the Canadian province of Alberta found an increase in cannabis-related ED presentations and calls to poison control between 2013 and 2019, shortly after the federal legalization of non-medical cannabis use (70). Furthermore, increases in CHS and unintentional ingestion of cannabis were documented over this period (70). Federal data collected as part of the electronic Canadian Hospitals Injury Reporting and Prevention Program (eCHIRPP) database found an overall 30.1% increase of cannabis-related cases between 2015 and 2018, though the overall rate of cannabis-related cases was relatively rare (71).

## Approaches to Minimizing Acute Harms: Recommendations for Public Health

One goal of cannabis legalization has been to prioritize public health by taking a harm reduction approach to regulating cannabis use (72), which conflicts with the prohibitionist model that has dominated cannabis legislation for decades (73). However, legalization without any restriction can be just as harmful to public health as prohibition (72, 73); thus, careful attention has to be paid to maximizing safety of the legal cannabis retail market. To this end, we highlight three specific areas relevant to minimizing acute harms that need to be considered when implementing cannabis legalization models: 1) early restriction of cannabis edibles and newer products for which less safety data are currently available; 2) proper labelling of cannabis products that clearly and consistently communicates dose/serving size information and health risks; and 3) a robust framework of data collection to monitor harms associated with cannabis use, which ideally should be broken down by consumer characteristics and product type to stratify risk.

### Early Restriction of Edibles and High-Potency Cannabis Products

The acute harms associated with use of alternative cannabis products (i.e., other than dried flower) are less known, but increasing evidence has suggested these harms might be a significant public health issue. Survey data from Colorado found that both trying new cannabis products and using cannabis edibles were associated with greater odds of experiencing an unexpected high, and that unexpected highs often led to acute psychological harms such as paranoia, panic attacks, hallucinations, and ED visits (33). In the year or so after legalization, Colorado saw a 63% increase in cannabis-related poison center calls for children, which was largely due to accidental cannabis edible ingestion (59). Colorado also saw four high-profile deaths related to consumption of edibles that occurred shortly after the legal retail market opened (60), and accumulating evidence suggested that edibles contributed to increased rates of cannabis-related ED visits (59, 60, 74). As a result of these harms, Colorado created a task force to address safety issues related to use of cannabis edibles, which resulted in tighter regulations and stricter packaging requirements (74).

These data strongly argue in favor of restriction of sales of edible products. However, complete prohibition of edible cannabis would undermine the success of legalization, as edibles are popular products that are prevalent even in jurisdictions without legal cannabis laws (74). As a result, the data from both Colorado and Washington state favor early restriction of edibles and high-potency cannabis products; this gives time for the retail market to stabilize and for data collection systems to be implemented, allowing for increased safety when newer cannabis products are eventually legalized (74). In addition, as there currently exists very little data to judge risks associated with the use of many of these newer cannabis products, delaying their sales in legal markets can allow for more time to conduct proper placebo-controlled safety trials.

### Proper Labelling of Products With Clear Information on Dose and Potential Harms

Evidence from multiple jurisdictions with a legal cannabis retail market has demonstrated that consumers often have very little understanding of product labelling information (75). For example, data collected as part of an online cross-sectional survey conducted among youth and young adults in Canada in October 2017 found that participants had limited understanding of quantitative THC labelling (76). In Canada, THC dose information is currently presented in a way that is likely confusing to consumers, i.e. displaying a “total THC amount” that includes both THC and its inactive acid precursor THCA, as well as a “THC amount” that excludes THCA (75). Another study that conducted focus groups in Colorado and Washington states in February 2016 found that consumers had limited familiarity with labels on edible products, and had difficulty interpreting doses expressed in mg (77). Confusion in Colorado state could come from the requirement to display a range of THC potencies to reflect variation in product testing (75). Consumers’ understanding of dose information can be even poorer for other types of cannabis products, such as oils that are expressed as mg THC per mL volume, which require greater numeracy skills (75).

The difficulty that consumers have in interpreting labels is compounded by factors such as the diversification of products; the same “dose” of THC is not necessarily comparable across different routes of administration (42, 75). This suggests that “dose expression” information may be needed so that consumers can compare serving sizes across different cannabis products (75), though it should be noted that this may not be entirely perfect given substantive differences in the pharmacokinetics of THC across routes of administration such as inhaled and oral (45). In addition, there are often few visual cues that signal the strength or potency of cannabis products, especially in the case of edibles, where one edible product can contain one or 20 “doses” of THC (75). Focus groups in Colorado and Washington state have demonstrated that even experienced users of cannabis edibles often cannot predict the degree of intoxication associated with edible use (44), which is likely exacerbated by unclear labelling and ineffective communication of dose.

To address the concern of effective labelling and communication of THC doses, a recent commentary by Hammond (75) outlined five specific issues to be addressed: clear labelling of cannabis products that requires minimal numeracy to understand; standardization of doses (or servings) of cannabis that does not exceed the typical amount required to become intoxicated; clarity of dose expression on labels; packaging that reinforces label information (e.g. unit-dose packaging); and labelling that can provide comparison between different products, to the extent possible. Other reviews have similarly emphasized the need for clearer labelling of serving size and dose information (59, 74). Other packaging-related issues have been raised, such as the need for packaging that deters children and has clear universal symbols to indicate that a product contains cannabis (59), and the need for consistent product testing to ensure that dose and potency information on labels is accurate (59, 78) For example, one analysis of cannabinoid content reported by state-certified laboratories in Washington state found significant variability between testing facilities, with some facilities consistently reporting higher or lower cannabinoid concentrations, likely due to systematic differences in testing methodology (78). Universal testing standards are needed to standardize dose and potency information on cannabis product labels.

In addition to providing clear information about dose and serving sizes, labels should convey health messages to inform consumers of the risks associated with cannabis use. Results of focus groups and surveys have been promising in suggesting that current cannabis users react positively towards the inclusion of health labels on cannabis products, and that health labelling may be effective in changing health-related behaviors (77, 79–81). For example, data collected as part of a survey of Canadians aged 16 to 30 years found that about 88% of respondents supported having health warnings on cannabis products, and that pictorial health warnings were perceived as more believable and effective than text-based warnings (79). Another online experimental study of university students in Alberta, Canada found that viewing cannabis packages with health warnings increased health knowledge (80). An analysis of data from the 2019 Global Drug Survey (a large international cross-sectional web-based survey) found that health labels may have the most impact among less frequent users of cannabis (81). However, an important caveat is that many consumers may not read product labels if there is too much information, as demonstrated in focus groups in Colorado and Washington (77), which supports the need for warnings that are either entirely pictorial or at least have minimal text.

Taken together, the existing data from Canada and the US strongly argue in favor of early efforts to standardize cannabis product labels with clear information that can be interpreted with minimal numeracy skills. To increase comprehension of dose and serving size information, there is increasing need to define a standard unit dose of THC, to indicate unit doses in a clear (i.e., non-numerical or minimally numerical) and consistent manner, and to apply this unit dose across products, to the extent possible. In addition, the use of pictorial health warnings on cannabis product labels has the potential to increase health knowledge and thus reduce acute harms associated with use. However, more research is needed to identify the most important messages in order to minimize the amount of information contained on a label. For example, participants in focus groups in Colorado and Washington state suggested that a link to a website for further information would be useful on cannabis labels (77). Having resources that consumers can use to find more safety information can help to minimize the scope of information required on product labels.

### Robust Data Collection to Monitor Harms Associated With Cannabis Products, by Consumer Characteristics and Product Type

A recurring theme in this review has been the scarcity of data on harms associated with newer and more potent cannabis products that are emerging in legal retail markets. Thus, proper infrastructure for robust collection of data on harms associated with cannabis is crucially important in any cannabis legalization model. In particular, data should be broken down by cannabis product type, potency, and route of administration and consumer characteristics such as age, sex, gender, and ethnicity, which will allow for stratification of risk. Multiple different types of data are required; for example, in addition to public health and safety data, market data (including information on sales, consumption, and possession) are vital to understanding how changes in regulatory approaches influence consumption patterns (74, 82). These data will likely come from multiple sources (e.g. reporting from licenced producers of cannabis, ED admissions, calls to poison control centers, federal/state/provincial surveys), but will need to be integrated by a single regulatory system to allow for monitoring of impact and performance of regulatory changes (74).

There are a number of challenges to integrating information from these data sources to monitor performance (83). One issue is the lag time between the implementation of policy changes and the availability of data, which results in delays in understanding changes in acute harms. Relatedly, existing sources of data (e.g. federal or state surveys) often do not collect detailed information on cannabis product information (quantity, potency, route of administration, etc.), and so adding in questions to address these issues takes time to implement. There can also be issues with hospital admissions or poison control data if there are not clear and consistent definitions of the relation of cases to cannabis use, though the quality of these data will improve over time with increasing data collection (83). One potential strategy to address some of these issues, as discussed by Young and colleagues (83), is the use of “social big data”, i.e. data from sources such as social media, portable/wearable devices (e.g. FitBit), and online search engines. While much of this vast quantity of available data exists as free-text entries (e.g. posts on social media) that would take a human researcher an impractically long time to analyze, the emerging use of machine learning has made this approach feasible in recent years (83).

## Conclusion

While legalization of non-medical cannabis use has the potential to improve public health and restore justice to the disadvantaged communities most impacted by cannabis prohibition, it also has the potential to increase harms in the absence of clear restrictions. Data emerging from Colorado, other US states, and Canada show that cannabis legalization has led to an increased potency and diversification of cannabis products, which in turn has been associated with increased risk of harms such as acute psychological distress, gastrointestinal and/or cardiovascular symptoms, cannabis-related injuries, and increased risk of ED visits. In order to mitigate these harms, future cannabis legalization models should incorporate three approaches: early restriction of cannabis edibles and high potency products; implementation of clear and effective labelling of cannabis products with both dose/serving size information and health risks; and integration of a robust data collection framework to monitor acute harms, including data broken down by consumer characteristics and product type to identify higher-risk populations and consumption patterns. The early restriction of cannabis edibles and other products will allow for the market to stabilize before introducing these higher-risk products, and will allow for more data collection to assess the extent of existing harms. While more data on product labelling are needed to find the right balance between clarity and scope of information, existing data suggest that the use of quantitative THC data alone can limit understanding, while the use of pictures and graphics improves label effectiveness and believability. Data collection and monitoring frameworks will need to take advantage of existing data sources such as hospitalizations, poison center calls, and federal or state surveys. In addition, there may be a role of “social big data”, e.g. using social media data to monitor trends and patterns in cannabis consumption and related harms in real time. The true impact of cannabis legalization on public health will not be known for quite some time. For now, the goal should be to continue collecting data and to learn from the jurisdictions that have already legalized non-medical cannabis use.

## Author Contributions

JM and BF: conception of the manuscript. JM: writing the first draft. All authors contributed to the article and approved the submitted version.

## Funding

This article was supported by a Mitacs Accelerate Award to BF in partnership with Canopy.

## Conflict of Interest

BF has obtained funding from Pfizer (GRAND Awards, including salary support) for investigator-initiated projects. He has some in-kind donation of cannabis product from Aurora and medication donation from Pfizer and Bioprojet and was provided a coil for TMS study from Brainsway. He has obtained industry funding from Canopy (through research grants handled by CAMH or University of Toronto), Bioprojet, ACS and Alkermes. He has received in kind donations of nabiximols from GW Pharma for past studies funded by CIHR and NIH. He is supported by a clinician scientist award by the Department of Family and Community Medicine of University of Toronto. JM is currently supported by a Mitacs grant obtained by BF in partnership with Canopy.
